# Patient and Observer Scar Assessment of Facial Keystone Flap Reconstruction in Korean Patients

**DOI:** 10.3390/jcm15010262

**Published:** 2025-12-29

**Authors:** Wooseob Kim, Eun A Jang, Kap Sung Oh, Kyu Nam Kim

**Affiliations:** Department of Plastic and Reconstructive Surgery, Kangbuk Samsung Hospital, Sungkyunkwan University School of Medicine, Seoul 03181, Republic of Korea; wskim2404@naver.com (W.K.); insane.blood.a@gmail.com (E.A.J.); kapsung.oh@samsung.com (K.S.O.)

**Keywords:** facial reconstruction, facial keystone flap, scars

## Abstract

**Background/Objectives**: Facial reconstruction using keystone flaps (KFs) provides reliable outcomes; however, few studies have evaluated postoperative scarring using patient-reported outcomes. We assessed scar outcomes after facial KF reconstruction using the Patient and Observer Scar Assessment Scale (POSAS) and explored associations with clinical and sociodemographic factors. **Methods**: In this retrospective observational cohort study, 43 Korean patients (27 males, 16 females; mean age: 49.95 ± 20.66 [range, 10–88] years) who underwent facial KF reconstruction between January 2020 and November 2022 were stratified by age (≤30, 40s–50s, ≥60 years), KF type (hemi-KF vs. other types), and sex. **Results**: Patients aged ≤30 years showed significantly higher total Patient Scar Assessment Scale (PSAS) scores (18.53 ± 5.69) than those aged 40s–50s (13.33 ± 3.27) and ≥60 years (11.82 ± 3.28) (*p* < 0.001). Observer Scar Assessment Scale and patient satisfaction scores did not significantly differ across flap types or sex; however, the hemi-KF group showed a trend toward better outcomes, and female patients reported higher PSAS scores. **Conclusions**: Facial KF reconstruction yields generally satisfactory scar outcomes. Younger patients are more critical of scars, and female patients exhibit greater aesthetic sensitivity. Patient-reported scar assessments are essential for individualized counseling, planning, and scar care in facial reconstruction.

## 1. Introduction

Facial defects are common in plastic surgery and require meticulous planning, even for small lesions [1,2]. As the face is the primary focus of aesthetics and function, reconstruction must balance both cosmetic and functional goals [1,2,3,4]. Local flaps remain the standard approach, often producing favorable outcomes [1,2,5]. However, additional scars from flap harvest sites may compromise the final aesthetic result [5,6,7]. Minimizing postoperative scarring is therefore essential in facial reconstruction, and scar assessment provides useful feedback for improving cosmetic outcomes [5,6,7]. Various local flap techniques—including Limberg, rhomboid, bilobed, V-Y, axial, and perforator-based flaps—are commonly used to address facial defects [1,2,5,8,9,10,11,12,13]. Scar outcomes have been evaluated using scales such as the Vancouver Scar Scale, Manchester Scar Scale, and the Patient and Observer Scar Assessment Scale (POSAS), with varying results [6,14,15,16,17].

Among these techniques, the keystone flap (KF) and its modifications are increasingly used for facial reconstruction, offering technical simplicity, broad applicability, and reproducibility with an easy learning curve. Studies have reported successful facial KF reconstruction with reliable and favorable outcomes [1,2,8,9]. However, only a few studies have focused on scar evaluation using patient-reported outcome measurements (PROMs) in facial KF reconstruction.

Accordingly, this study aimed to evaluate postoperative scar outcomes following facial KF reconstruction in Korean patients using POSAS, focusing on clinical and sociodemographic influences.

## 2. Materials and Methods

### 2.1. Ethical Approval and Study Design

The study protocol and research process adhered to the ethical guidelines of the 1975 Declaration of Helsinki. The Institutional Review Board of Kangbuk Samsung Hospital approved this study (approval number: 2023-08-009). Written informed consent was obtained from all patients. All patients consented to provide their information and photos for use in online open-access publications before undergoing treatment procedures and surgeries.

In this observational cohort study, we retrospectively examined Korean patients who underwent KF reconstruction for facial defect coverage between January 2020 and November 2022. Patients treated with skin grafts or other local flaps were excluded. Clinical data—including age, sex, defect cause and location, defect and flap size, flap type, complications, follow-up duration, and POSAS score—were extracted from electronic medical records, operation logs, and electronic clinical photographs.

### 2.2. Surgical Techniques—Facial Defect Coverage with Modified KF Techniques

**Figure 1:**
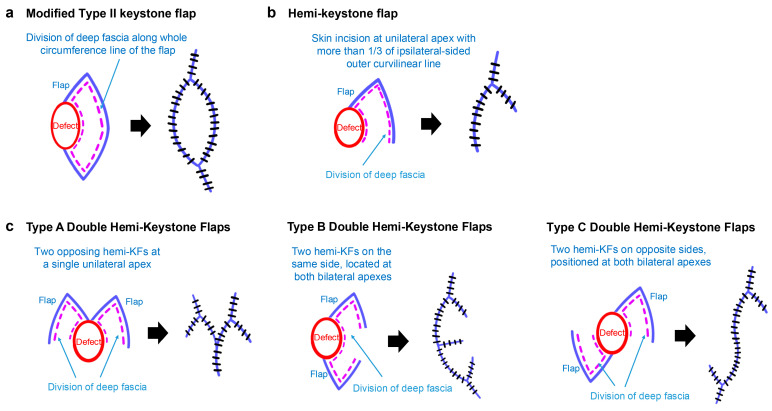
Schematic illustrations of the modifications of keystone flap (KF) techniques used in this study. (**a**) Modified Type II KF technique. (**b**) Hemi-KF technique. (**c**) Double hemi-KFs techniques: Type A, Type B, and Type C.

We applied three modified KF types for facial defect coverage as follows: the modified Type II KF, which involves division of the fascial layer (release of the superficial musculoaponeurotic system) along the entire flap perimeter [9,18,19]; the hemi-KF, a partial KF involving a skin incision with fascial layer division on one side of the flap apex, extending less than half of the outer curvilinear line of the flap [20,21,22]; and the double hemi-KFs, comprising two hemi-KFs arranged in three distinct patterns: Type A features two opposing hemi-KFs at a single unilateral apex; Type B includes two hemi-KFs on the same side, at both ipsilateral apexes; and Type C comprises two hemi-KFs on opposite sides, at both contralateral apexes (Figure 1).

Regarding KF modification to cover each facial defect, we tried to follow a modified stepwise algorithm devised in our previous study (Supplementary Figure S1) [18]. Supplementary Figure S1 presents a stepwise approach for modified KF reconstruction. If a defect can be reliably covered with a single hemi-KF, it is used. If not, an additional hemi-KF is added, considering tissue laxity and relaxed skin tension lines (RSTLs). Based on flap arrangement, Type A, B, or C double hemi-KFs are applied. If further flap movement with secure vascularity is needed, the modified Type II KF is used. This algorithm guides flap selection based on defect characteristics. Doppler devices were not routinely used to locate skin perforator hot spots because the face is considered a well-vascularized area with abundant perforators.

When designing the flap after the final defect was created, we considered the following points. First, the flap was designed to be slightly larger than the defect in areas with sufficient tissue laxity [1,8,9,18]. Second, the flap axis was constructed to be as parallel to the facial relaxed skin tension lines as possible, which could facilitate favorable scar formation by decreasing wound tension [1,2,8]. Third, flap outlines (incision lines) were asymmetrically designed and positioned either within or along facial subunits whenever possible, to enhance scar appearance by distracting from postoperative scars and providing a visual illusion of normal skin contours [1,2,8]. Fourth, the use of multiple flaps (double hemi-KFs) was considered on a case-by-case basis to achieve effective recruitment of surrounding tissue laxity.

In all KF modifications, the deep fascial layer (the facial superficial musculoaponeurotic system) was divided along the skin incision, and minimal flap undermining was performed to preserve flap vascularity via central perforators [9,18,19]. The sequence of flap insetting was as follows: the defect side flap was initially sutured, followed by V Y advancement closure at the flap apex and final closure of the flap donor site.

### 2.3. Postoperative Scar Management

All patients received standardized scar care: microporous tape for 1 month post-suture removal, followed by 3 months of scar ointment and silicone gel sheet therapy.

### 2.4. Postoperative Scar Assessment

POSAS, a widely used scar-specific PROM, was used to assess scar burden and quality through two six-item, 10-point scales: the Patient Scar Assessment Scale (PSAS) and the Observer Scar Assessment Scale (OSAS) [14,23,24]. The PSAS, completed by the patient, evaluates six scar characteristics: pain, itching, color, stiffness, thickness, and irregularity [24,25]. The OSAS, completed by the observer, comprises six items: vascularity, pigmentation, thickness, pliability, relief, and surface area [23,24]. Total scores range between 6 and 60, with 6 representing perfectly normal skin and 60 indicating the worst imaginable scar [23,24]. In addition to PSAS and OSAS scores, each patient and observer provided a general opinion of scar appearance (the patient-reported overall satisfaction score and the observer-reported overall scar rating), rated on a 1–10 scale (1 and 10 corresponding to the best and worst possible scar appearance, respectively) [23,24]. The PSAS in this study was completed by each patient at the final follow-up. The OSAS was conducted by two independent physicians (K.S.O. and K.N.K.), and the average of their scores was used to minimize the risk of bias.

### 2.5. Data Collection and Patient Cohort Groups

Data were anonymized and analyzed using Microsoft Excel (Microsoft, Redmond, WA, USA). We performed statistical analysis of the POSAS outcomes according to patient age, KF type, and sex. First, patients were classified into three groups according to age. Patients in their 30s or younger, 40s to 50s, and 60s or older were categorized into groups A, B, and C, respectively. Next, we classified patients who underwent defect coverage with hemi-KF and other types of KF (modified Type II KF and double hemi-KF) into groups D and E, respectively. Lastly, we classified male and female patients into groups F and G, respectively.

### 2.6. Statistical Analysis

#### 2.6.1. Software and Basic Statistics

We used R language version 3.3.3 (R Foundation for Statistical Computing, Vienna, Austria) and STATA version 15 (StataCorp, College Station, TX, USA) software for all statistical analyses. Continuous and categorical variables are expressed as mean ± standard deviation (SD) and sample number and percentage (N [%]), respectively.

#### 2.6.2. Assessment of Normality of Distribution of Continuous Variables

The Shapiro–Wilk test was used to assess the normality of the distribution of continuous variables.

#### 2.6.3. Comparison of the Mean Difference in Paired Variables

The differences between paired variables in groups A, B, and C are expressed as mean ± SD. The Kruskal–Wallis H test was used to evaluate differences among these groups, and Bonferroni tests were applied for post hoc analysis. The differences between groups D and E are also presented as mean ± SD and were analyzed using Student’s *t*-test and the Mann–Whitney U test. Similarly, the differences between groups F and G are reported as mean ± SD and were analyzed using the same statistical tests. The significance level was set at *p* < 0.05.

## 3. Results

Table 1 summarizes patient demographics and clinical data. A total of 43 patients (27 male and 16 female patients), with an average age of 49.95 ± 20.66 (range, 10–88) years, were enrolled in this study. Defect causes were diverse, including trauma, skin tumors, and fistulas. The mean value of defect sizes was 3.64 ± 2.96 cm^2^. All defects were successfully covered using the modified KF techniques. The mean value of flap sizes was 5.63 ± 6.88 cm^2^. Hemi-KF was used for 26 defects, double hemi-KF for 11 defects, and modified Type II KF for 6 defects. No major complications were reported in the study cohort. The distal flap tip maceration in one case healed within 1 week of conservative dressing. The mean follow-up period was 7.34 ± 2.85 (range, 4–15) months.

Comparing groups classified according to ages, the mean values of the total PSAS scores in groups A (n = 17), B (n = 15), and C (n = 11) were 18.53 ± 5.691, 13.33 ± 3.266, and 11.82 ± 3.281, respectively (*p* < 0.001). Post hoc analysis showed group A > group B and C. The mean value of each PSAS item, except for thickness, showed significant differences between groups A and B and between groups A and C. The mean values of the overall patient satisfaction scores in groups A, B, and C (3.76 ± 1.480, 2.80 ± 0.862, and 2.82 ± 0.874, respectively) showed no significant between-group difference (*p* < 0.074). The mean values of the total OSAS scores in groups A, B, and C (17.29 ± 3.759, 17.97 ± 4.011, and 15.41 ± 4.061, respectively) showed no significant between-group difference (*p* < 0.093). The mean value of each OSAS item, except for surface area, showed no significant between-group difference. The mean values of the objective scar rating in groups A, B, and C (3.56 ± 0.846, 3.77 ± 0.904, and 3.09 ± 1.221, respectively) showed no significant between-group difference (*p* < 0.063) (Table 2).

Comparing groups classified by KF type, the mean values of the total PSAS scores in groups D (n = 26; 14.73 ± 6.004) and E (n = 17; 15.41 ± 3.825) showed no significant between-group difference (*p* < 0.337). No PSAS items showed significant between-group differences. The mean values of the overall patient satisfaction scores in groups D (3.08 ± 1.197) and E (3.35 ± 1.272) showed no significant between-group difference (*p* < 0.466). The mean values of the total OSAS scores in groups D (16.54 ± 3.658) and E (17.82 ± 4.387) showed no significant between-group difference (*p* < 0.304). No OSAS items showed significant between-group differences. The mean values of the objective scar rating in groups D (3.42 ± 0.913) and E (3.65 ± 1.101) showed no significant between-group difference (*p* < 0.587) (Table 3).

Comparing groups classified by sex, the mean values of the total PSAS scores in groups F (n = 27; 13.96 ± 4.109) and G (n = 16; 16.75 ± 6.445) showed no significant between-group difference (*p* < 0.110). No PSAS items showed significant between-group differences. The mean values of the overall patient satisfaction scores in groups F (2.93 ± 1.207) and G (3.63 ± 1.147) showed no significant between-group difference (*p* < 0.124). The mean values of the total OSAS scores in groups F (17.48 ± 3.531) and G (16.31 ± 4.629) showed no significant between-group difference (*p* < 0.356). The mean value of each OSAS item, except for relief, showed no significant between-group difference. The mean values of the objective scar rating in groups F (3.52 ± 0.882) and G (3.50 ± 1.169) showed no significant between-group difference (*p* < 0.072) (Table 4).

Representative clinical cases of KF reconstruction are shown in Supplementary Figures S2–S4.

## 4. Discussion

The face plays a central role in both appearance and social interaction, making its reconstruction a high priority in plastic surgery [1,2,8,25,26]. In this study, we evaluated postoperative scarring after facial KF reconstruction using POSAS in Korean patients. To the best of our knowledge, this is the first PROM-based study on facial KF reconstruction.

Local flaps are ideal for small-to-moderate facial defects, offering like-with-like tissue for optimal color and texture match while avoiding donor site morbidity and improving cosmetic outcomes [1,2,8]. Despite these advantages, local flap reconstruction inevitably creates additional donor site scars, which may affect the final aesthetic result. Scarring concerns are often heightened among Asian patients—including Koreans—compared with Caucasians, largely due to distinct skin characteristics such as a thicker dermis, higher collagen density, increased melanin content, and greater sebaceous activity [27,28,29,30]. These features contribute to stronger fibroproliferative responses, prolonged inflammation, and a higher risk of hyperpigmentation and hypertrophic scarring [27,29].

Therefore, postoperative scar evaluation and management are especially important in Asian patients undergoing facial flap reconstruction [29]. Our PROM-based data may serve as a valuable tool for preoperative counseling and managing patient expectations. All patients in our study received standardized scar care: tape fixation for 1 month followed by combined therapy using scar ointment and silicone gel sheets for 3 months.

POSAS, developed by Draaijers et al. in 2004, is a validated tool for scar assessment that combines both patient (PSAS) and observer (OSAS) perspectives [23,24]. Given the subjective nature of scar perception, both components are essential. In this study, postoperative scars were evaluated using POSAS, with patient scores analyzed first according to age groups. Age is known to influence wound healing and scar formation; however, existing evidence remains inconclusive [31,32,33]. During the fetal growth period, humans exhibit diminished scarring and scarless healing patterns [34], and clinically, surgeons have observed that older adults tend to develop less exuberant scarring than younger individuals [31,32,33]. Although aging skin heals more slowly, it often results in less prominent scarring, which has been attributed to lower levels of stromal-derived factor 1, a mediator that is more abundant in younger individuals and associated with thicker scars [32].

Based on this biological and clinical background, patients were stratified into three age groups representing younger (≤30 years), middle-aged (40s–50s), and older (≥60 years) populations. Grouping patients in their 40s and 50s was intended to establish a middle-aged reference group between younger and older cohorts, consistent with prior literature suggesting distinct wound healing dynamics and scar perception across these life stages. Accordingly, we hypothesized that POSAS scores would be higher in younger patients and lower in older patients.

The total PSAS scores and all PSAS item scores, except for thickness, showed significant differences between groups A and B and between groups A and C. Thickness and overall patient satisfaction scores showed no significant differences; however, both trended toward higher (worse) scores in younger patients and lower (better) scores in older ones. The total OSAS scores, as well as all OSAS items except surface area, and objective scar rating showed no significant group differences. Only surface area, reflecting scar extent, differed significantly between groups.

While age-related scar formation is important scientifically, scar appearance and sensation are inherently subjective. The PSAS results likely reflect higher aesthetic standards in younger patients. OSAS results by age may reflect differences between actual scar formation and observer perception. As assessed by two plastic surgery experts, these scores provide an objective reference for facial KF scar outcomes. Only the OSAS “surface area” item showed a significant age-related difference. Other items, though not significant, trended toward higher (worse) scores in younger groups and lower (better) scores in older groups. These findings highlight the need for thorough preoperative scar counseling, especially in younger individuals undergoing facial KF reconstruction.

We analyzed POSAS scores by KF type. As more incisions can lead to more noticeable scars [35,36], we hypothesized that hemi-KFs—requiring fewer incisions—would yield better outcomes. However, PSAS and OSAS scores did not differ significantly between flap types. This finding suggests that other factors, such as skin type, color, creases, and relaxed skin tension lines, may also affect scar appearance [1,2,37]. Nonetheless, our observation of lower PSAS, OSAS, and satisfaction scores in group E suggests that hemi-KFs may result in better scar outcomes. Our stepwise use of modified KF techniques may provide a cost-effective approach to achieving favorable aesthetic results.

We also analyzed POSAS scores by sex. Men generally have thicker, oilier skin, while women tend to have lighter pigmentation; however, hormonal factors and cosmetic preferences may influence both scar formation and perception [38,39,40,41]. In this study, POSAS scores showed no significant sex differences, but mean PSAS scores were higher in women, suggesting less favorable scar assessments. Among OSAS items, only “relief” (scar elevation/depression) scores differed significantly between men (2.76 ± 0.698) and women (2.59 ± 0.898, *p* < 0.027). Other items showed no significant sex differences, though the mean scores for each item were higher in men, indicating worse scar evaluations. These results align with previous findings: women often have stricter cosmetic standards, while men may experience more pronounced scar features due to their skin properties [41]. These differences reinforce the importance of personalized preoperative counseling, particularly for patients with heightened sensitivity to scar aesthetics.

This study has several important limitations. First, as a retrospective, single-center study with a relatively small sample size (n = 43), its statistical power is limited. Subgroup analyses stratified by age, sex, and flap type further reduce power and increase the risk of Type II error. Accordingly, findings described as trends—such as potentially favorable outcomes with hemi-keystone flaps or higher PSAS scores in female patients—should be interpreted as exploratory rather than confirmatory. Second, heterogeneity in defect location, size, etiology, and involved facial aesthetic units introduces potential confounding factors that could influence scar formation and patient perception. These variables could not be fully controlled in this retrospective cohort and may limit generalizability. Third, higher PSAS scores observed in younger patients may reflect not only greater aesthetic sensitivity but also biological differences in wound healing, including stronger fibroproliferative responses characteristic of younger Asian skin. Similarly, sex-related differences may be influenced by inherent skin properties rather than perception alone. Fourth, although the POSAS is a widely accepted PROM, the use of additional tools such as the SCAR-Q may allow more comprehensive assessment of psychosocial impact [42]. Finally, given that approximately 40% of facial scars improve within 3 months and early scar status may predict outcomes [43], the wide follow-up range (4–15 months) and lack of time-specific PROMs in this study limit meaningful longitudinal assessment of scar maturation. Future prospective, multicenter studies with larger cohorts and standardized follow-up intervals are needed to validate these findings.

Despite these limitations, this study is the first to use POSAS for scar evaluation in facial KF reconstruction based on PROMs. All procedures were performed by a single surgeon, contributing to procedural consistency and providing a meaningful foundation for future research. Looking ahead, the integration of artificial intelligence (AI) into clinical decision-making may offer valuable support in flap selection and surgical planning. A recent study has demonstrated the potential of large language models in supporting clinical reasoning [44]. In the context of facial reconstruction, AI-assisted systems may help optimize flap choice based on defect characteristics, patient-specific variables, and predicted outcomes. Further research into AI-driven tools could complement surgeon expertise and enhance personalized reconstructive strategies.

In conclusion, we assessed postoperative scarring after facial KF reconstruction using POSAS. Younger patients demonstrated higher PSAS scores, indicating greater subjective concern regarding scarring. Although no statistically significant differences were observed according to flap type or sex, hemi-KFs showed descriptively lower scar scores, and female patients tended to report higher PSAS scores. These observations should be interpreted cautiously and regarded as exploratory trends that warrant further investigation in larger, adequately powered studies.

Facial KFs do not inherently result in poor scarring. With proper preoperative counseling and postoperative care, including possible touch-up treatments, aesthetic concerns can be effectively managed. These findings may aid patient–surgeon decision-making and support future research into KF use beyond the face.

## Figures and Tables

**Table 1:** Summary of patient demographics and clinical characteristics.

Characteristics	Values
Total participants, *n*	43
Male sex, *n* (%)	27 (62.79)
Female sex, *n* (%)	16 (37.21)
Age, y, mean (SD)	49.95 ± 20.66
Defect cause	n (%)
Trauma	30 (69.76)
Skin tumor	10 (23.25)
Skin fistula	3 (6.97)
Defect location	n (%)
Dorsal subunit of the nasal unit	10 (23.25)
Central subunit of the forehead unit	6 (13.95)
Zygomatic subunit of cheek unit	5 (11.62)
Lateral subunit of the upper lip unit	4 (9.30)
Lateral canthal subunit of the eyelid unit	2 (4.65)
Lateral subunit of the cheek unit	2 (4.65)
Philtrum subunit of the upper lip unit	2 (4.65)
Lateral subunit of the forehead unit	2 (4.65)
Tip subunit of the nasal unit	2 (4.65)
Medial subunit of the cheek unit	2 (4.65)
Eyebrow subunit of the forehead unit	1 (2.32)
Lower lid subunit of the eyelid unit	1 (2.32)
Tip and dorsal subunit of the nasal unit	1 (2.32)
Buccal subunit of the cheek unit	1 (2.32)
Mental unit	1 (2.32)
Auricular unit	1 (2.32)
Defect size, cm^2^, mean (SD)	3.64 ± 2.96
Flap size, cm^2^, mean (SD)	5.63 ± 6.88
Flap types	n (%)
Hemi-keystone flap	26 (60.46)
Double hemi-keystone flap	11 (25.58)
Modified Type II keystone flap	6 (13.95)
Complications	n (%)
Infection	0
Dehiscence	1 (2.32)
Hematoma	0
Flap failure	0
Follow-up periods, months, mean (SD)	7.34 ± 2.85

**Table 2:** Comparison of Patient Scar Assessment Scale and Observer Scar Assessment Scale scores among age-stratified groups.

PSAS	Group A (Mean ± SD)	Group B (Mean ± SD)	Group C (Mean ± SD)	*p*-Value	Post Hoc Analysis
Pain	2.35 ± 1.169	1.40 ± 0.507	1.27 ± 0.467	0.001	A > B, C
Itching	2.71 ± 1.312	1.47 ± 0.640	1.64 ± 0.674	0.004	A > B
Color	3.53 ± 1.663	2.67 ± 0.724	1.73 ± 0.786	0.001	A, B > C
Stiffness	3.29 ± 1.047	2.53 ± 0.743	2.18 ± 0.603	0.003	A > B, C
Thickness	3.12 ± 0.993	2.60 ± 0.828	2.55 ± 0.934	0.129	A > C
Irregularity	3.53 ± 1.068	2.67 ± 0.976	2.45 ± 0.934	0.020	A > C
Total score	18.53 ± 5.691	13.33 ± 3.266	11.82 ± 3.281	0.001	A > B, C
Overall patient satisfaction score	3.76 ± 1.480	2.80 ± 0.862	2.82 ± 0.874	0.074	NA
**OSAS**	**Group A (Mean ± SD)**	**Group B (Mean ± SD)**	**Group C (Mean ± SD)**	***p*-Value**	**Post Hoc Analysis**
Vascularity	2.50 ± 0.829	2.70 ± 1.014	2.14 ± 0.234	0.339	NA
Pigmentation	2.53 ± 0.819	2.87 ± 1.288	2.18 ± 0.337	0.253	NA
Thickness	3.18 ± 0.749	3.37 ± 0.812	3.18 ± 1.079	0.403	NA
Pliability	2.82 ± 0.749	2.93 ± 0.799	2.55 ± 0.820	0.311	NA
Relief	2.76 ± 0.752	2.73 ± 0.799	2.55 ± 0.820	0.572	NA
Surface area	3.50 ± 0.729	3.37 ± 0.855	2.82 ± 1.007	0.034	A > C
Total score	17.29 ± 3.759	17.97 ± 4.011	15.41 ± 4.061	0.111	NA
Objective scar rating	3.56 ± 0.846	3.77 ± 0.904	3.09 ± 1.221	0.063	NA

Groups A, B, and C included patients aged ≤30 years, those in their 40s to 50s, and those aged ≥60 years. Differences between groups (A, B, and C) are presented as the mean ± standard deviation (SD). The Kruskal–Wallis H test was employed to assess overall group differences, and post hoc comparisons were conducted using Bonferroni-adjusted pairwise tests. A *p*-value of <0.05 was considered significant. Abbreviations: Patient Scar Assessment Scale (PSAS), Observer Scar Assessment Scale (OSAS).

**Table 3:** Comparison of Patient Scar Assessment Scale and Observer Scar Assessment Scale scores between groups stratified by flap type.

PSAS	Group D (Mean ± SD)	Group E (Mean ± SD)	*p*-Value
Pain	1.92 ± 1.129	1.47 ± 0.514	0.186
Itching	2.00 ± 1.233	2.00 ± 0.935	0.693
Color	2.65 ± 1.198	2.94 ± 1.638	0.655
Stiffness	2.62 ± 1.061	2.94 ± 0.748	0.132
Thickness	2.65 ± 1.018	3.00 ± 0.791	0.088
Irregularity	2.88 ± 1.107	3.06 ± 1.088	0.517
Total score	14.73 ± 6.004	15.41 ± 3.825	0.337
Overall patient satisfaction score	3.08 ± 1.197	3.35 ± 1.272	0.466
**OSAS**	**Group D (Mean ± SD)**	**Group E (Mean ± SD)**	***p*-Value**
Vascularity	2.48 ± 0.922	2.47 ± 0.649	0.364
Pigmentation	2.58 ± 1.046	2.53 ± 0.819	0.611
Thickness	3.08 ± 0.717	3.50 ± 0.984	0.176
Pliability	2.67 ± 0.706	2.97 ± 0.874	0.308
Relief	2.60 ± 0.788	2.85 ± 0.745	0.291
Surface area	3.13 ± 0.672	3.50 ± 1.104	0.233
Total score	16.54 ± 3.658	17.82 ± 4.387	0.304
Objective scar rating	3.42 ± 0.913	3.65 ± 1.101	0.587

Group D comprised patients who underwent defect coverage using a hemi-keystone flap (KF), whereas group E comprised those who underwent coverage with other KF types (modified Type II KF or double hemi-KF). Differences between groups (group D vs. group E) are presented as the mean ± standard deviation (SD). Student’s *t*-tests and Mann–Whitney U tests were applied to assess statistical differences between the groups. A *p*-value of <0.05 was considered significant. Abbreviations: Patient Scar Assessment Scale (PSAS), Observer Scar Assessment Scale (OSAS).

**Table 4:** Comparison of Patient Scar Assessment Scale and Observer Scar Assessment Scale scores between groups stratified by sex.

PSAS	Group F (Mean ± SD)	Group G (Mean ± SD)	*p*-Value
Pain	1.48 ± 0.580	2.19 ± 1.276	0.472
Itching	1.81 ± 0.962	2.31 ± 1.302	0.796
Color	2.70 ± 1.463	2.88 ± 1.258	0.288
Stiffness	2.67 ± 0.832	2.88 ± 1.147	0.065
Thickness	2.59 ± 0.694	3.13 ± 1.204	0.052
Irregularity	2.70 ± 0.993	3.38 ± 1.147	0.245
Total score	13.96 ± 4.109	16.75 ± 6.445	0.110
Overall patient satisfaction score	2.93 ± 1.207	3.63 ± 1.147	0.124
**OSAS**	**Group F (Mean ± SD)**	**Group G (Mean ± SD)**	***p*-Value**
Vascularity	2.59 ± 0.832	2.28 ± 0.774	0.473
Pigmentation	2.76 ± 1.050	2.22 ± 0.657	0.501
Thickness	3.26 ± 0.685	3.22 ± 1.095	0.179
Pliability	2.81 ± 0.709	2.75 ± 0.913	0.079
Relief	2.76 ± 0.698	2.59 ± 0.898	0.027
Surface area	3.03 ± 0.800	3.25 ± 1.017	0.045
Total score	17.48 ± 3.531	16.31 ± 4.629	0.356
Objective scar rating	3.52 ± 0.882	3.50 ± 1.169	0.072

Groups F and G comprised male and female patients, respectively. Differences between groups (Group F vs. Group G) are presented as the mean ± standard deviation (SD). Student’s *t*-tests and Mann–Whitney U tests were performed to assess statistical differences between the groups. A *p*-value of <0.05 was considered significant.

## Data Availability

The original contributions presented in this study are included in the article/Supplementary Material. Further inquiries can be directed to the corresponding author(s).

## Supplementary Materials

The following supporting information can be downloaded at: https://www.mdpi.com/article/10.3390/jcm15010262/s1, Figure S1: Modified algorithm for the stepwise application of the modified KF technique, Figure S2: Clinical photographs of nasal defect coverage with hemi-KF, Figure S3: Clinical photographs of preauricular defect coverage with double hemi-KFs, Figure S4: Clinical photographs of upper lip defect coverage with modified Type II KF.

## Abbreviations

The following abbreviations are used in this manuscript:

KF	Keystone flap
POSAS	Patient and Observer Scar Assessment Scale
PSAS	Patient Scar Assessment Scale
PROM	Patient-reported outcome measurement
OSAS	Observer Scar Assessment Scale
SD	Standard deviation

## References

[1] Yoon C.S., Kim H.B., Kim Y.K., Kim H., Kim K.N. (2019). Relaxed skin tension line–oriented keystone–designed perforator island flaps considering the facial aesthetic unit concept for the coverage of small to moderate facial defects. Medicine.

[2] Lim S.Y., Yoon C.S., Lee H.G., Kim K.N. (2020). Keystone design perforator island flap in facial defect reconstruction. World J. Clin. Cases.

[3] Kim Y.J., Park J.W., Kim J.M., Park S.H., Hwang J.H., Kim K.S., Lee S.Y., Shin J.H. (2013). The functionality of facial appearance and its importance to a Korean population. Arch. Plast. Surg..

[4] Yıldız T., Selimen D. (2015). The impact of facial aesthetic and reconstructive surgeries on patients’ quality of life. Indian J. Surg..

[5] Rao J.K., Shende K.S. (2016). Overview of local flaps of the face for reconstruction of cutaneous malignancies: Single institutional experience of seventy cases. J. Cutan. Aesthet. Surg..

[6] Vaidya T.S., Mori S., Khoshab N., Dusza S.W., Bander T., Matros E., Rossi A.M., Nehal K.S., Lee E.H. (2019). Patient-reported aesthetic satisfaction following facial skin cancer surgery using the FACE-Q Skin Cancer Module. Plast. Reconstr. Surg. Glob. Open.

[7] Woodard C.R. (2013). Complications in facial flap surgery. Facial Plast. Surg. Clin. N. Am..

[8] Lee H.G., Kong Y.T., Kim K.N. (2021). Use of keystone flaps in consideration of the facial aesthetic subunit concept as an alternative reconstructive option for nasal defect coverage. J. Craniofac. Surg..

[9] Yoo B.W., Oh K.S., Kim J., Shin H.W., Kim K.N. (2023). Modified keystone perforator island flap techniques for small- to moderate-sized scalp and forehead defect coverage: A retrospective observational study. J. Pers. Med..

[10] Agrawal N.K. (2021). Revisiting rhombic flaps for aesthetic facial resurfacing: Addressing a surgical conundrum. J. Cutan. Aesthet. Surg..

[11] Gualtieri M., Scivoletto G., Pitino F., D’Archivio L., Valentini V. (2025). Limberg flap for lateral midface reconstruction. J. Craniofac. Surg..

[12] Salgarelli A.C., Cangiano A., Sartorelli F., Bellini P., Collini M. (2010). The bilobed flap in skin cancer of the face: Our experience on 285 cases. J. Craniomaxillofac. Surg..

[13] Lombardo G.A., Tamburino S., Tracia L., Tarico M.S., Perrotta R.E. (2016). Lateral nasal artery perforator flaps: Anatomic study and clinical applications. Arch. Plast. Surg..

[14] Raklyar E., Zloty D.M. (2012). Use of a patient and observer scar assessment scale to evaluate the V-Y advancement flap for reconstruction of medial cheek defects. Dermatol. Surg..

[15] Ellabban M.A., Ibrahim A.M., Gomah A.A., Salah O., Abdelrahman I., Steinvall I., Adly O.A., Aboelnaga A.M. (2021). Assessment of freestyle local facial perforator flaps for coverage of facial defects. J. Craniofac. Surg..

[16] Gulati A., Grekin R., Neuhaus I., Saylor D., Yu S., Park A., Seth R., Knott P.D. (2023). Long-term appearance-related outcomes of facial reconstruction after skin cancer resection. Facial Plast. Surg. Aesthet. Med..

[17] Peters F., Mücke M., Möhlhenrich S.C., Bock A., Stromps J.P., Kniha K., Hölzle F., Modabber A. (2021). Esthetic outcome after nasal reconstruction with paramedian forehead flap and bilobed flap. J. Plast. Reconstr. Aesthet. Surg..

[18] Kim K.H., Yoo B.W., Lim S.Y., Oh K.S., Kim J., Shin H.W., Kim K.N. (2022). Modified keystone perforator island flap for tension-reducing coverage of axillary defects secondary to radical excision of chronic inflammatory skin lesions: A retrospective case series. BioMed Res. Int..

[19] Yoon C.S., Kong Y.T., Lim S.Y., Kim J., Shin H.W., Kim K.N. (2021). A comparative study for tension-reducing effect of type I and type II keystone perforator island flap in the human back. Sci. Rep..

[20] Petukhova T.A., Navrazhina K., Minkis K.V.-Y. (2020). V-Y Hemi-keystone advancement flap: A novel and simplified reconstructive modification. Plast. Reconstr. Surg. Glob. Open.

[21] Hifny M.A., Park T.H. (2022). Customized reconstruction with rotation Hemi-Keystone flap. J. Cosmet. Dermatol..

[22] Park T.H. (2022). Aesthetic reconstruction of auricular keloids with a novel hemi-keystone flap. Aesthet. Plast. Surg..

[23] Draaijers L.J., Tempelman F.R.H., Botman Y.A.M., Tuinebreijer W.E., Middelkoop E., Kreis R.W., van Zuijlen P.P.M. (2004). The patient and observer scar assessment scale: A reliable and feasible tool for scar evaluation. Plast. Reconstr. Surg..

[24] van de Kar A.L., Corion L.U.M., Smeulders M.J.C., Draaijers L.J., van der Horst C.M.A.M., van Zuijlen P.P.M. (2005). Reliable and feasible evaluation of linear scars by the patient and observer scar assessment scale. Plast. Reconstr. Surg..

[25] Dayan S., Clark K., Ho A.A. (2004). Altering first impressions after facial plastic surgery. Aesthet. Plast. Surg..

[26] Singh P., Birkett L., Dhar S., Krumhuber E., Mosahebi A., Ponniah A. (2024). Facial beauty and the correlation of associated attributes: An empirical aesthetic database study. Plast. Reconstr. Surg. Glob. Open.

[27] Sykes J.M. (2007). Management of the aging face in the Asian patient. Facial Plast. Surg. Clin. N. Am..

[28] McCurdy J.A. (2007). Considerations in Asian cosmetic surgery. Facial Plast. Surg. Clin. N. Am..

[29] Kim S., Choi T.H., Liu W., Ogawa R., Suh J.S., Mustoe T.A. (2013). Update on scar management: Guidelines for treating Asian patients. Plast. Reconstr. Surg..

[30] Shirakabe Y., Suzuki Y., Lam S.M. (2003). A new paradigm for the aging Asian face. Aesthet. Plast. Surg..

[31] Marcus J.R., Tyrone J.W., Bonomo S., Xia Y., Mustoe T.A. (2000). Cellular mechanisms for diminished scarring with aging. Plast. Reconstr. Surg..

[32] Nishiguchi M.A., Spencer C.A., Leung D.H., Leung T.H. (2018). Aging suppresses skin-derived circulating SDF1 to promote full-thickness tissue regeneration. Cell Rep..

[33] Colboc H., Meaume S., Téot L., Mustoe T.A., Middelkoop E., Gauglitz G.G. (2020). Chapter 44: Scar and Scarring in the Elderly. Textbook on Scar Management: State of the Art Management and Emerging Technologies.

[34] Larson B.J., Longaker M.T., Lorenz H.P. (2010). Scarless fetal wound healing: A basic science review. Plast. Reconstr. Surg..

[35] Han S.-E., Ryoo S.-T., Lim S.Y., Pyon J.-K., Bang S.-I., Oh K.-S., Mun G.-H. (2013). Minimizing surgical skin incision scars with a latex surgical glove. Aesthet. Plast. Surg..

[36] Veldhuizen I.J., Brouwer P., Aleisa A., Kurtansky N.R., Dusza S.W., Nehal K.S., Hoogbergen M.M., van der Hulst R.R.W.J., Lee E.H. (2022). Nasal skin reconstruction: Time to rethink the reconstructive ladder?. J. Plast. Reconstr. Aesthet. Surg..

[37] Wallace H.J., Fear M.W., Crowe M.M., Martin L.J., Wood F.M. (2017). Identification of factors predicting scar outcome after burn in adults: A prospective case-control study. Burns.

[38] Rahrovan S., Fanian F., Mehryan P., Humbert P., Firooz A. (2018). Male versus female skin: What dermatologists and cosmeticians should know. Int. J. Womens Dermatol..

[39] Kim H.I., Kwak C.Y., Kim H.Y., Yi H.S., Park E.J., Kim J.H., Park J.H. (2018). Correlation between dermal thickness and scar formation in female patients after thyroidectomy. Arch. Craniofac. Surg..

[40] Kundu K., Rawat V.S., Chattopadhyay D. (2021). Gender differences in quality of life and psychological impact of facial burn scars in a tertiary care center. Burns.

[41] Rankin M., Borah G.L. (2003). Perceived functional impact of abnormal facial appearance. Plast. Reconstr. Surg..

[42] Ziolkowski N.I., Pusic A.L., Fish J.S., Mundy L.R., Wong She R., Forrest C.R., Hollenbeck S., Arriagada C., Calcagno M., Greenhalgh D. (2020). Psychometric findings for the SCAR-Q patient-reported outcome measure based on 731 children and adults with surgical, traumatic, and burn scars from four countries. Plast. Reconstr. Surg..

[43] Shao K., Taylor L., Miller C.J., Etzkorn J.R., Shin T.M., Higgins H.W., Giordano C.N., Sobanko J.F. (2021). The natural evolution of facial surgical scars: A retrospective study of physician-assessed scars using the patient and observer scar assessment scale over two time points. Facial Plast. Surg. Aesthet. Med..

[44] Marcaccini G., Seth I., Xie Y., Susini P., Pozzi M., Cuomo R., Rozen W.M. (2025). Breaking Bones, Breaking Barriers: ChatGPT, DeepSeek, and Gemini in Hand Fracture Management. J. Clin. Med..

