# Supplementary material for: Patient and Observer Scar Assessment of Facial Keystone Flap Reconstruction in Korean Patients
Source: J Clin Med. 2025 Dec 29;15(1):262. doi: 10.3390/jcm15010262 (PMC12787151; doi:10.3390/jcm15010262)
Supplement: Supplementary file 1 [file jcm-15-00262-s001.zip › jcm-4065408-supplementary.docx]

**Supplementary Information**

**
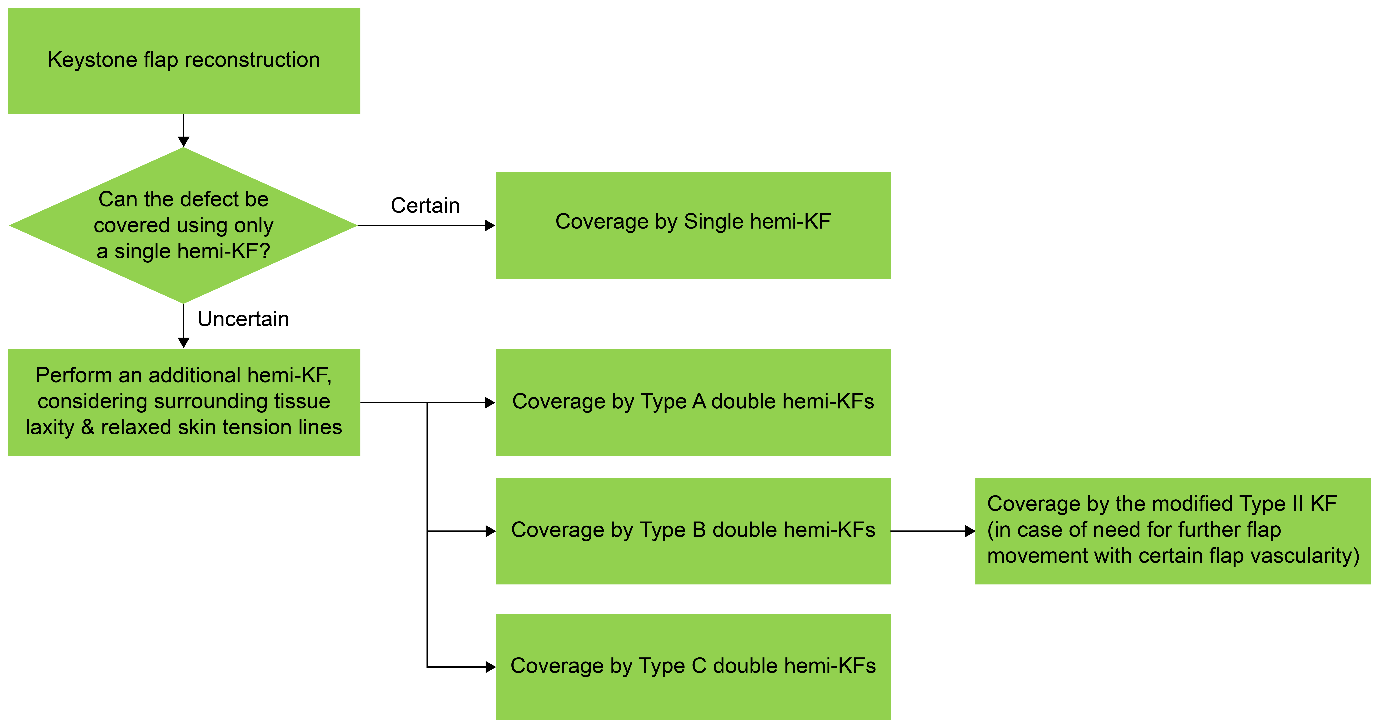
**

**Supplementary Figure S1**. Modified algorithm for the stepwise application of the modified KF technique.


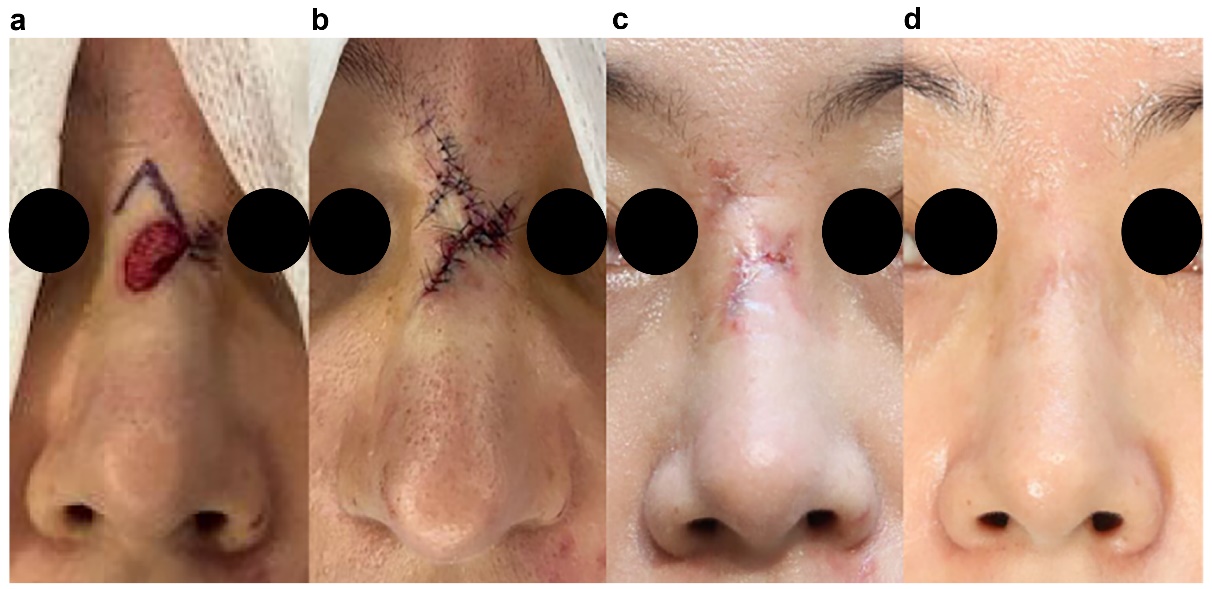


**Supplementary Figure S2**. Clinical photographs of nasal defect coverage with hemi-KF. (A) Skin defect (1 × 1.5 cm²) due to trauma on the dorsal subunit of the nasal unit and design of a hemi-KF (1 × 1 cm²). (B) Successful flap coverage. (C) One-month follow-up photograph. (D) At the last follow-up, 12 months postoperatively, the total Patient Scar Assessment Scale (PSAS) score, Observer Scar Assessment Scale (OSAS) score, patient satisfaction score, and objective scar rating were 13, 9, 3, and 2, respectively.


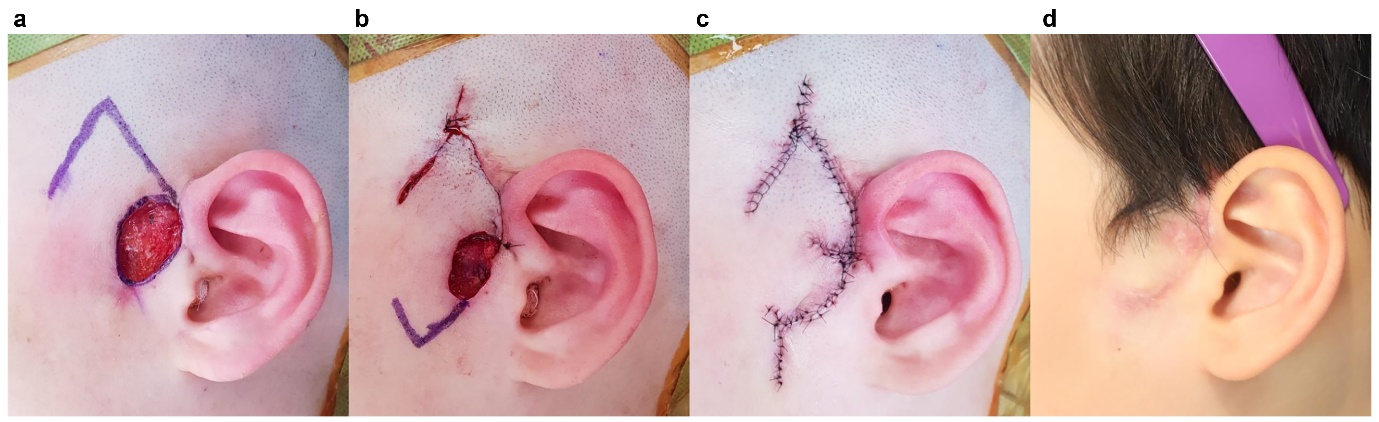


**Supplementary Figure S3**. Clinical photographs of preauricular defect coverage with double hemi-KFs. (A) Skin and soft tissue defect (1.5 × 2 cm²) caused by wound dehiscence following preauricular fistulectomy to the zygomatic subunit of the left cheek unit. Initial design of a hemi-KF (3 × 3 cm^2^) on the upper side of the defect. (B) Design of an additional hemi-KF (1.5 × 2 cm^2^) on the lower side of the defect. (C) Successful flap coverage. (D) At the last follow-up, 9 months postoperatively, the total PSAS score, OSAS score, patient satisfaction score, and objective scar rating were 18, 23, 3, and 4.5, respectively.


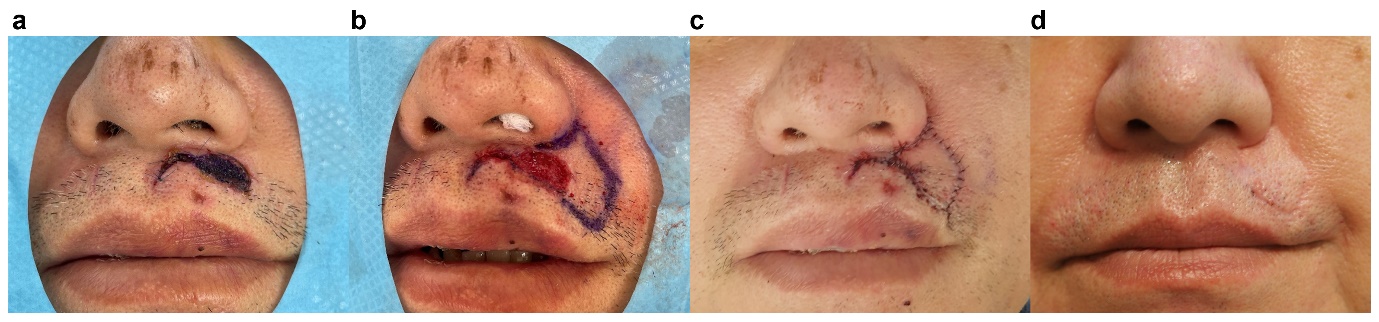


**Supplementary Figure S4**. Clinical photographs of upper lip defect coverage with modified Type II KF. (A) Skin and soft tissue defect (1.5 × 2 cm²) due to trauma on the lateral subunit of the left upper lip. (B) Design of a modified Type II KF (1.5 × 3 cm²). (C) Successful flap coverage. (D) At the last follow-up, 11 months postoperatively, the total PSAS score, OSAS score, patient satisfaction score, and objective scar rating were 11, 14.5, 2, and 3, respectively.
